# Review of biosensing with whispering-gallery mode lasers

**DOI:** 10.1038/s41377-021-00471-3

**Published:** 2021-02-26

**Authors:** Nikita Toropov, Gema Cabello, Mariana P. Serrano, Rithvik R. Gutha, Matías Rafti, Frank Vollmer

**Affiliations:** 1 Department of Physics and Astronomy, Living Systems Institute, University of Exeter, Exeter, EX4 4QD UK; 2 Departamento de Química, Instituto de Investigaciones Fisicoquímicas Teóricas y Aplicadas, Universidad Nacional de La Plata, La Plata, 1900 Argentina

**Keywords:** Imaging and sensing, Biophotonics, Optical sensors

## Abstract

Lasers are the pillars of modern optics and sensing. Microlasers based on whispering-gallery modes (WGMs) are miniature in size and have excellent lasing characteristics suitable for biosensing. WGM lasers have been used for label-free detection of single virus particles, detection of molecular electrostatic changes at biointerfaces, and barcode-type live-cell tagging and tracking. The most recent advances in biosensing with WGM microlasers are described in this review. We cover the basic concepts of WGM resonators, the integration of gain media into various active WGM sensors and devices, and the cutting-edge advances in photonic devices for micro- and nanoprobing of biological samples that can be integrated with WGM lasers.

## Introduction

Lasers have played a crucial role in optics since Theodore Maiman first reported on *Stimulated Optical Radiation in Ruby* 60 years ago^1^. Experiments with lasers have enabled the development of quantum optics theory^2^, as well as many different applications, for example, in manufacturing, imaging, spectroscopy, metrology and sensing^3^. During the last few years, the application of microlasers, especially whispering-gallery mode (WGM) microlasers, in chemical and biological sensing has increased due to advances made in reducing the gap between laboratory experiments and their real-world application. A number of exciting applications of WGM lasers in biosensing have been reported, such as lasing within living cells^4^, monitoring contractility in cardiac tissue^5^, detection of molecular electrostatic changes at biointerfaces^6^, label-free detection of single virus particles^7^ and advancement of in vivo sensing^4,8,9^. WGM microlasers with a liquid-core optical ring resonator (LCORR) can probe the properties of a gain medium introduced as a fluid into the core of a thin-walled glass capillary, thereby providing good sensitivity for the detection of health biomarkers such as DNA and protein molecules^10,11^.

Many optical platforms are potentially useful for label-free sensing in biology and chemistry. Examples include optical sensors that make use of plasmonic nanostructures and nanoparticles, photonic crystals, tapered optical fibres, zero-mode waveguides and passive WGM resonators^12–18^. These micro- and nanoscale optical platforms have already been used for some of the most demanding biosensing tasks such as detection of single molecules^17,19^ and detection of single influenza A virus particles and other nanoparticles^20,21^. The use of WGM microlasers for chemical and biological sensing can offer sensing modalities that are often not easily accessed on other optical sensor platforms. For example, in vivo sensing with WGM microlasers is facilitated by detection of the emission of relatively bright laser light at frequencies that are spectrally well separated from the frequency of the free-space excitation beam. Furthermore, WGM microlasers may offer a potentially very high detection sensitivity for molecules due to the narrow linewidth of the laser lines, which could enable detection of the frequency shifts induced by single molecules. Herein, we aim to provide a comprehensive review of the emerging field of biological and biochemical sensing with WGM microlasers. Our review is structured as follows. In the first section, we review the building blocks of WGM microlaser devices, their biosensing applications and their sensing mechanisms. In the second and third sections, we focus on the use of WGM microdroplet resonators in biosensing and the most recent advances made in the integration of WGM microdroplet resonators with gain media. In the fourth section, we review state-of-the-art techniques for micro- and nanoprobing of biological samples that can be combined with WGM microlasers. We close with a discussion of the prospects of using emerging WGM microlasers in biological and chemical sensing applications and as an emerging research tool for single-molecule biosensing.

### WGM microlasers in biosensing

#### Building blocks

Similar to conventional lasers, WGM microlasers consist of three principal building blocks: the gain medium, the pump source, and the optical resonator—here the WGM resonator. The gain medium defines most of the spectral, temporal and power characteristics of the laser light emission. Usually, an optical pump source supplies the energy needed to maintain population inversion of the active particles in the gain medium, i.e., fluorophore molecules, for light amplification by stimulated emission of radiation. The various gain media that have already found use in WGM microlaser-based sensors are reviewed in sections ‘Microdroplet resonators as active cavities in biosensing’ and ‘Review of gain media in WGM microlasers for sensing’.

The performance of a WGM microlaser can be further characterised by the quality factor (Q-factor) of the optical resonator, which is a measure of the damping of the resonator modes. The Q-factor is defined as the ratio of the stored energy to the energy dissipated per radian of the oscillation. Various important laser parameters depend on the Q-factor of the cavity such as (i) the laser linewidth, a measure of the spectral coherence of the laser emission and its monochromaticity; (ii) the photon lifetime, the time it takes for the energy in the resonator cavity to decay to 1/*e* of the original value; and (iii) the lasing threshold, the lowest optical pump power at which stimulated emission is observed. Several optical waves (modes) can typically be excited in a WGM resonator; the separation between two neighbouring modes is called the free spectral range. Another important parameter for resonators is the finesse^22^, which is the free spectral range of the cavity modes divided by the linewidth (FWHM) of the resonances. The finesse corresponds to the number of roundtrips the light takes inside a WGM microcavity before the stored energy decays to 1/*e* of the original value.

The term whispering-gallery wave was first used by Lord Rayleigh to describe the propagation of sound waves in the dome of St Paul’s Cathedral^23^; whispering-gallery wave or mode is now used to describe the effect of any wave travelling around a concave surface. Comparable to this effect, light in a WGM microlaser is confined through near-total internal reflection and circumnavigates a typically spherical cavity, such as a glass microbead^24^; the interference of the light results in WGM optical resonances. WGMs may be confined in cavity geometries such as disks^25^, toroids^26^, or deformed hexagonal resonators^27^. The unique characteristics of WGM microcavities, such as the long lifetime of the intracavity photons and the small volume of the modes, make them excellent candidates for constructing WGM microlasers with low lasing thresholds that exhibit narrow spectral linewidths. To the best of our knowledge, the first WGM laser was made from a highly polished crystalline calcium fluoride (CaF_2_) sphere of 1–2 mm diameter. The rare-earth ion samarium (Sm^2+^) was used as the optical gain dopant^28^. Since then, lasing has been demonstrated in many different spherical WGM cavity geometries^8,28,29^ and others, such as triangular nanoplatelets^30^ and ZnO hexagonal and dodecagonal microrods and nanonails^31^. Achieving a low lasing threshold is especially important in biosensing applications where the photodamage of biological samples must be avoided. The light intensities of WGMs range from MWcm^−2^ to GWcm^−2^ and are comparable to those in microscopy^32^. For example^22^, for a WGM resonator of 40 µm diameter and finesse 3 × 10^5^, when the input power is 16 µW, the built‐up circulating optical power is as high as 800 mW, and the circulating optical intensity is 20 MWcm^−2^. WGM lasing thresholds at µW pump power levels and below have been demonstrated^33–35^. Lasing thresholds of nJ have been shown for spatially and temporally incoherent optical pumping^36^. WGM thresholds of µJ cm^−2^ have been demonstrated for pumping with a pulsed laser^37^. Apart from optical pumping with a pulsed laser, the ability to realize free-space continuous-wave optical pumping^38^ is advantageous because it allows for a wider selection of wavelengths, a smaller laser linewidth and thus a potentially higher sensitivity in biosensing applications.

#### Sensing mechanisms

In Table 1, we list WGM microlasers that have been used in some of the most exciting and most recent sensing and biosensing applications. In this section, we review some of the basic sensing mechanisms and how they have been used in the respective applications.

According to the Shawlow-Townes formula, the linewidth of WGM microlasers with cold-cavity Q-factors of 10^8^ pumped at 1550 nm using erbium as a gain medium can reach a few hertz, thus rendering WGM lasers potentially useful in biosensing applications that require a high detection sensitivity^39–41^. For example, microsphere or microring resonators doped with a gain material can provide a 10^4^-fold narrower resonance linewidth than a passive microcavity^42^. Changes in the refractive index in the surrounding medium cause spectral shifts of the WGMs, which can be used for detecting biomolecules. A spectral shift of the lasing line on the order of ∆*ω* = 210.8 kHz corresponds to refractive index changes on the order of 10^−9^. The detection of a very low concentration of biomolecules becomes possible if these spectral WGM shifts are resolved^42^. Another interesting WGM sensing modality uses the changes in the effective linewidth, where resonance broadening is attributed to the stress-induced mode shift of different polar modes in the emission spectrum of fluorescent dye-doped 6–10 µm diameter polystyrene microspheres. These changes have been used to monitor the forces that deform these microspheres when they are engulfed by a cell during the biological process endocytosis^43^.

The interaction of a nanoparticle with the evanescent field of a WGM can lift the degeneracy of the clockwise and counterclockwise propagating modes, resulting in mode splitting^44^. Monitoring the frequency shifts of the laser lines from WGM splitting is a mechanism that has been used for highly sensitive detection of nanoparticles, down to nanoparticles ~15 nm in radius^45,46^. The WGM frequency shifts due to mode splitting are typically too small to be resolved directly on a spectrometer. Instead, the frequency splitting is measured by recording the beat note that is produced when an ultranarrow emission line of a WGM microlaser is split into two modes by a nanoparticle scatterer. The mode-splitting sensing mechanism with WGM microlasers has been applied in biosensing for detection of ~120 nm influenza A virus particles deposited on the WGM sensor from air using an erbium-doped silica microtoroid cavity^7^.

Another interesting sensing mechanism for WGM microsphere lasers makes use of the concepts of Förster resonance energy transfer (FRET) and coherent radiative energy transfer (CRET)^47^. Following these mechanisms, the WGM microlaser doped with donor molecules can exhibit changes in its emission intensity and wavelength upon surface binding of acceptor molecules. WGM cavities composed of liquid crystal (LC) droplets and doped with donor/acceptor molecules were used to detect the FRET signals in the emission spectrum of the droplets. WGM microlaser LC droplets were used to detect FRET signals of fluorophores such as rhodamine B isothiocyanate and rhodamine-phycoerythrin as they attached to the LC droplet surface^47^.

#### WGM sensing platforms

The most versatile WGM microlasers provide true platforms for the development of various applications in chemical and biological sensing. One of these platforms is the so-called optofluidic ring resonator-based dye laser^48^. This microfluidic dye laser is based on a liquid-core optical ring resonator. The LCORR is made of a fused silica capillary with a wall thickness of a few microns. The circular cross-section of the capillary forms a ring resonator that supports WGMs and provides optical feedback for lasing, for example, by injecting a dye solution. Due to the high Q-factor of the WGM, a low lasing threshold can be achieved for pulsed laser excitations of ~1 μJ mm^−2^. A large fraction of the mode intensity extends into the capillary, where a gain medium such as rhodamine B can be introduced. LCORR lasers have been employed in a range of biosensing applications. In one example, the indocyanine green (ICG) fluorophore was dissolved in blood plasma and then injected into the LCORR capillary to demonstrate sensing in blood. When injected into blood, ICG binds primarily to plasma proteins and lipoproteins, resulting in enhanced fluorescence and lasing^10^. In a similar optofluidic ring resonator approach, a single layer of DNA molecules was used to provide laser gain in DNA detection^49^. Intercalating DNA dyes were employed so that there was no lasing from nontarget DNA on this digital DNA detection platform^49^. Gong et al.^50^ recently introduced the concept of a distributed fibre optofluidic laser. Due to precise fibre geometry control via fibre drawing, a series of identical optical microcavities uniformly distributed along a hollow optical fibre (HOF) was achieved to obtain a one-dimensional distributed fibre optofluidic laser. An enzymatic reaction catalysed by horseradish peroxidase was monitored in the HOF over time, and changes in the product concentration were measured by laser-based arrayed colourimetric detection. The fabricated five-channel detection scheme is shown in Table 1. In general, optofluidic resonator platforms combine the merits of a low-threshold lasing with ultranarrow WGM lasing spectra and microfluidic integration. Furthermore, they are suitable for switching between single- and multimode lasing regimes, and they provide optofluidic tuneability of the lasing wavelength^51^.

Another interesting sensing platform that solves the problem of fabricating WGM microlasers with different sensing specificities was demonstrated by coating passive ‘microgoblet’ WGM cavities with multifunctional molecular inks^52^. The one-step modification process uses dip-pen lithography to coat the passive ‘goblet’ cavity with phospholipid inks to introduce optical gain and provide molecular binding selectivity at the same time. The ink was applied such that it solely coated the light-guiding circumference of a prefabricated polymer ‘goblet’ microresonator. The authors showed that the highly localised deposition of the ink suffices for low-threshold lasing in air and water. In air, the observed lasing threshold was ∼10 nJ per pulse, which is only approximately three times that demonstrated in similar goblet microlasers where the entire volume was dye-doped. The authors demonstrated biosensing applications, for example, detecting streptavidin binding to biotin that was contained in the ink and provided molecular binding selectivity. Streptavidin binding to the microcavity was detected from a redshift in the WGM laser mode^52^.

Another WGM microlaser platform that is versatile and may find more widespread use comprises the demonstration of ultrasound modulation of the laser output intensity of WGM microdroplets; this platform may enable the development of laser emission-based microscopy for deep tissue imaging^53^.

A liquid crystal biosensor platform based on WGM lasing has been reported for real-time and high-sensitivity detection of acetylcholinesterase (AChE) and its inhibitors^54^. The spectral responses provide direct information about molecular adsorption/desorption at the liquid crystal/aqueous solution interface and can be used as an indicator of enzymatic reactions. The limit of detection achieved was as low as 0.1 pg mL^−1^ for fenobucarb and 1 pg mL^−1^ for dimethoate, which is considerably lower than the standard levels of the pesticides specified for water quality standards. The results indicated that this versatile platform has potential for application in real-time and highly sensitive monitoring of biochemical reactions.

Ouyang et al.^55^ presented an optofluidic chip platform that was integrated with directly printed, high-Q polymer WGM microlaser sensors for ultrasensitive enzyme-linked immunosorbent assay (ELISA). It was demonstrated that such an optofluidic biochip can measure horseradish peroxidase (HRP)–streptavidin, which is a widely used catalytic molecule in ELISA, via chromogenic reaction at a concentration of 0.3 ng mL^−1^. Moreover, it enables on-chip optofluidic ELISA of the disease biomarker vascular endothelial growth factor (VEGF) at the extremely low concentration of 17.8 fg mL^−1^, which is over 2 orders of magnitude better than the ability of current commercial ELISA kits.

**Table 1:** WGM laser-based biosensors and their physical sensing mechanisms

WGM microlaser/sensor	Device example	Application	Sensing/lasing mechanism
Lasing-encoded microsensor driven by interfacial cavity energy transfer^47^		Molecular barcoding	Lasing upon binding: CRET, FRET
WGM laser with a rhodamine 6G gain medium^42^		Refractive index change measurements	Wavelength shift and intensity changes
Bioelectrostatic sensitive droplet lasers (liquid crystals)^6^		Ultrasensitive label-free biosensing and monitoring of molecular interactions	Wavelength shift under molecular electrostatic changes
Optofluidic lasers (optofluidic ring resonator, OFRR)^10,11^		Intracavity bio/chemical sensing, biocontrolled photonic devices, and biophysics	Combination of FRET with highly nonlinear optofluidic lasers
			Fluorophore laser emission for detecting albumins, globulins, and lipoproteins in blood
Distributed fibre optofluidic laser^50^		Enzymatic reaction catalysed by horseradish peroxidase monitoring	Laser-based arrayed colourimetric detection
Lasing within live cells containing intracellular optical microresonators^4,56^		Barcode-type cell tagging and tracking, intracellular sensing, adaptive imaging, and labelling	Uptake of a fluorescent WGM microbead laser into the cell cytoplasm, i.e., by endocytosis
Self-heterodyned microlasing^7^		Individual nanoparticle detection in real time and single virus detection in air	Beat-note signal at radiofrequency
Coumarin 6-doped polystyrene microspheres in cells^43^		Direct measurement of the biomechanical stress induced by a live cell during endocytosis	Broadening and blueshift of the resonances due to deformation of the microbead partially incorporated by a cell
Ultrasound modulated droplet lasers^53^		Microscopy for deep tissue imaging	Laser emission-based microscopy
Optofluidic laser with DNA molecules as gain materials^49^		Target DNA detection with a single laser pulse, in a digital manner	DNA produces laser emission, while the fluorescence background is suppressed
Optofluidic biolasers^57^		Intracavity and biochemical analysis	Amplification of small concentration differences in the gain medium
Intracellular lasers^8^		Cytoplasmic internal stress and its dynamic fluctuations; tagging	Determination of alterations in multiple spectral peaks
Biointegrated microlasers in cardiac tissue^5^		Monitoring contractility in cardiac tissue	Multimode lasing and frequency shift
Egg white and chicken albumen-based biological microlasers^58,59^		Medical treatments, biotracking, biosensing, and bioimaging	Not presented
Liquid crystal biosensor based on WGM lasing^54^		Real-time and high-sensitive detection of AChE and its inhibitors	Spectral responses to molecular adsorption/desorption
On-chip integrated polymer WGM microlaser sensor^55^		Ultrasensitive enzyme-linked immunosorbent assay (ELISA)	Lasing changes

## Microdroplet resonators as active cavities in biosensing

To achieve high Q-factors, which are important for sensing and lasing applications, WGM resonators require smooth, near-spherical surfaces that limit scattering losses. Recently, water-walled cavities have been explored to provide an ultrasmooth cavity surface and more than 10^6^ recirculation cycles of light^60^. This work points to an important class of devices that can be easily and inexpensively made with microdroplets. In the following section, we review the application of WGM microdroplet resonators in biosensing.

### WGM microdroplets for lasing

Droplet resonators in liquid–air or liquid–liquid configurations achieve high quality factors and finesse because they can trap light via near-total internal reflection using the ultrasmooth liquid interface. Liquids with a higher refractive index, such as oils or aqueous glycerol solutions, are desirable for the miniaturisation of devices and for minimising radiation losses. For example, recent sensing approaches use high-refractive-index oil to make droplet resonators with a high Q-factor up to 1.6 × 10^7^ ^61,62^.

Air–liquid microdroplets suitable for WGMs are easy to create by using water or other liquids. A high surface tension naturally forms water droplets in air. The surface tension of water in droplet form is 8000 times stronger than gravity. In the case of liquid–liquid droplets, immiscibility of the two components is often required and is an advantage in sensing. The liquids are self-contained, with minimum cross-contamination, which provides good biocompatibility and often longevity for the sensing device^8,63^. The most common method to make droplets is to use a dispenser with a sharp tip such as a syringe and slowly push the liquid out into another liquid or on top of a solid surface. The surface tension of the drop will help it maintain its position, and the amount of liquid pushed out will determine the droplet diameter^8,54,64^. Some reports have also used the natural drying of liquids until tiny droplets are formed because of the surface tension^65,66^. Finally, the cavity resonances of the WGMs can be manipulated by controlling the surface tension around the droplets, either by streaming the background fluid or by stretching the droplet using a dual-beam trap^64,67^.

The requirements for the occurrence of stimulated emission with a certain frequency in WGM lasers entail excitation of a gain medium by a pump source, confinement of the resultant light and feedback from the microcavity. The droplet cavity medium can be easily mixed with molecules and submicron particles, such as fluorescent particles, biomolecules or specific binding chemical molecules. These will act as a gain medium and can later be used for light emission from the droplets and for various sensing mechanisms based on WGM droplet lasing^8,64,66,68,69^.

Droplet-based WGM microlasers can be advantageous in sensing and biosensing; compared to other WGM resonator structures, they offer very high Q-factors that can exceed 10^9^ under ideal conditions because of their naturally smooth surfaces as a result of surface tension^70^. Such high-Q resonant modes allow lasing at very low threshold pump powers. In addition, the position of the droplets inside a given medium can be controlled using optical trapping, magnetic fields, electrodynamic ion traps (Paul traps) and ultrasonic waves^69,71–75^. Droplet-based resonators with liquid crystals as an integral component within the droplet offer an opportunity for tuning and tailoring droplet properties, such as the orientation of the LCs using electric fields^6,54^. Tuning of the droplet-based sensor can improve its sensitivity limits and makes it capable of sensing biomolecules with negative charges^6^. However, fabrication and tuning of LC droplets is complicated; therefore, their use for in vivo applications has not yet been demonstrated.

### WGM droplet microlasers in sensing and biosensing

Yang et al. demonstrated reconfigurable liquid droplets by dispensing a solution of dichloromethane and epoxy resin using a computer-controlled microplotter^64^. Due to adhesion, a tiny drop of the liquid was left hanging on the outside wall of the dispenser after the dispenser was immersed in the solution. The dispenser was then touched to the soap water surface, which pulled the drop of solution down to the soap water surface, and the drop self-assembled into a circular floating microdroplet due to the surface tension of water and the high viscosity and immiscibility of the epoxy. The size of the droplet was controlled by the dispenser size and the immersion depth when the dispenser touched the epoxy solution. The formation of self-assembled droplets was demonstrated by Duong Ta et al.^65^ They prepared a polymer solution composed of polystyrene, dichloromethane and epoxy resin and dipped a metal rod with a sharp tip inside it. The tip was then immersed into a PDMS solution and moved parallel at a constant speed until the solution completely left the tip. This created a fibre shape of the solution on the PDMS with decreasing diameter from the point where the metal rod touched the PDMS to where it left. Because of the high surface tension of the epoxy resin, the fibre spontaneously broke into numerous small pieces, forming well-aligned spherically shaped droplets. These liquid droplet microlasers are particularly exciting for biosensing applications because they have demonstrated excellent biocompatibility and miniature sizes^8,63,68,76,77^.

Although droplet-based WGM microlasers may offer many advantages over other microcavities, they also face some challenges, such as deformation and evaporation because of their volatile nature, mechanical instabilities because of weak binding forces, low WGM coupling efficiencies and problems related to their positioning. To overcome the problem of toxicity, naturally occurring materials, such as lipids and starch granules, have been explored for producing WGM droplets and microlasers for biosensing^8,63,78,79^.

## Review of gain media in WGM microlasers for sensing

### Fluorescent dyes

Fluorescent dyes are common gain materials in WGM microlasers for sensing and biosensing. Fluorophores can usually provide better biocompatibility than quantum dots (QDs), which, depending on their composition, are often toxic^80^. Some dye molecules, such as indocyanine green (ICG) and fluorescein, are of special interest in this regard because they have been approved by the US Food and Drug Administration for human use^81^. Other dyes, such as cypate, rhodamine 110, Oregon green and Tokyo green, are also claimed to be noncytotoxic with a wide range of experimental data in support of this^70^.

Various dye-doped droplets have been used to demonstrate lasing from WGM-based microlasers. They have found some important applications in the imaging, labelling and tracking of cells because of the ease of implanting them within cells and because of their biocompatible nature^4,8^. WGM microlasers doped with rhodamine, coumarin 6, coumarin 102, ICG, or Nile red have been used to detect temperature, stress, water vapour and various biological molecules, such as bovine serum albumin (BSA) and acetylcholinesterase^4,8,54,63,64,66,76,82^. Most organic fluorescent dyes suffer from photobleaching, which restricts repetitive measurements and the use of high pump powers to enhance the signal unless the fluorescent material is regenerated^83^. One of the possible ways to address this problem is the replacement of dyes with polymers^84^. For example, an optofluidic microlaser with an ultralow threshold down to 7.8 µJ cm^−2^ in an ultrahigh‐Q WGM microcavity filled with a biocompatible conjugated polymer has been demonstrated^85^. This conjugated polymer exhibits a significant enhancement in lasing stability compared with Nile red. Polymer microspheres can be used as biomarkers or assay substrates in chemical diagnostics, flow cytometry and biological imaging.

### Fluorescent biomaterials

Fluorescent biomaterials naturally occurring in living organisms, such as flavin mononucleotide^86^, *Gaussia* luciferase^63^, green fluorescent protein^63,87^, Venus yellow fluorescent protein^68^, firefly luciferin^88^ and chlorophyll^89^, have been merged into droplets and other resonator structures as the gain media for WGM microlasers. Researchers^58^ demonstrated that natural egg white is an excellent biomaterial for a WGM laser cavity. Using a simple dehydration method, dye-doped goose egg white microspheres were obtained with various sizes from 20 to 160 µm in diameter. These microspheres can act as laser sources under optical excitation with a lasing threshold of ~26 µJ mm^−2^ and a Q-factor up to 3 × 10^3^. Another example of the use of natural materials for lasing is chicken albumen^59^. These microsphere biolasers can operate in aqueous and biological environments such as water and human blood serum, which makes them promising candidates for laser-based biosensing and biological applications.

Higher excitation powers are usually required for these biomaterials to initiate lasing compared to organic dyes because of their low quantum yields^86^. Most importantly, due to their nonsynthetic origin, fluorescent biomaterials show promise for in vivo sensing applications (see the section ‘WGM microlaser-based sensors in living systems: extra- and intracellular sensing’).

### Rare-earth elements

Rare-earth elements have been used in many examples as the gain medium in WGM microlasers. Stimulated emission has been demonstrated with WGM cavities with samarium^28^, as well as neodymium, erbium, thulium, and holmium^90^. Erbium is an interesting gain dopant that can be applied in a sol–gel process to fabricate WGM microlasers. Yang et al.^91^ reported on erbium-doped and Raman microlasers on a silicon chip fabricated by the sol–gel process, where Q-factors as high as 2.5 × 10^7^ at 1561 nm were obtained. Ions of Er^3+^ can be used to achieve lasing in different spectral bands. Er-doped TiO_2_ thin films grown by the sol–gel technique can demonstrate sharp emission peaks at 525 nm, 565 nm, 667 nm, and 1.54 µm^92^.

Er:Yb-doped glass WGM microlasers have been demonstrated by using a CO_2_ laser to melt Er:Yb glass onto silica microcapillaries or fibres^93^. This proposed WGM structure facilitates thermo-optical tuning of the microlaser modes by passing gas through the capillary and can be used for sensing, such as anemometry. The same group reported anomalous pump-induced lasing suppression in Yb:Er-doped microlasers^94^. Usually, a pump source achieves lasing in a system, and in most cases, a stronger pump leads to higher laser power at the output. However, in this case, the authors observed that this behaviour may be suppressed if two pump beams are used.

WGM sensing mechanisms are based on tracking the resonance shift or Q-factor spoiling, monitoring WGM intensity changes^95^, and using photon upconversion^96^. WGM-modulated green and red upconversion with a Q-factor up to 45,000 was achieved in a 9 μm Er:Yb codoped tellurite sphere located in methanol^97^. The authors assessed its application in refractometric sensing and its advantages for the detection of nanoparticles with a diameter of <50 nm. Refractometric sensing with a detection sensitivity of 7.7 nm/RIU was demonstrated.

Although several sensing applications of active WGM cavities doped with rare-earth ions have been demonstrated, their use in biosensing is limited because a high pump power is often required for lasing, especially for upconversion lasing^98^.

### Quantum dots

Quantum dots are a common gain material in WGM microlasers for sensing^66,99^. Quantum dots are colloidal or epitaxial semiconductor nanocrystals in which the electron–hole pair is confined in all three spatial dimensions. They are characterised by tuneable emission wavelengths, high quantum yields, and resistance to photobleaching^99^. Laser emission into modes of a dielectric microsphere has been observed using different QDs, such as optically pumped HgTe QDs on the surface of a fused silica microsphere^100^ or semiconductor ZnO hexagonal nanodisks^101^.

The temperature dependence of the resonant wavelengths of a WGM microbottle doped with CdSe QDs has been studied^102^. These WGM resonators exhibit a blueshift with increasing temperature. It has been observed that these shifts are linear with temperature over an ~10 nm wavelength range. This system has been found to be highly photostable for temperature sensing applications.

Another example of QDs in WGM lasers is core-shell CdSe/ZnS QDs, which can be embedded in polystyrene microspheres^99^. Their potential for targeted biosensing was explored through the addition of a protein that adsorbs to the microsphere surface, thrombin, and one that does not, bovine serum albumin. Such sensors demonstrate an approximately 100 nm/RIU sensitivity and have interesting advantages such as remote excitation and remote sensing^103^. WGM resonators doped with CdSe/ZnS QDs have also been used to demonstrate the concept of automatic label-free WGM sensing of alcohol in water and of bacterial spores in water^104^.

An interesting example is silicon QDs, which are especially attractive for fluorescent refractometric sensors because of their low toxicity and ease of handling^105^. The authors^105^ showed that silica microspheres with a thin layer of Si QDs immersed in a cuvette with methanol demonstrate WGM resonance shifts as a function of the refractive index of the analyte solution, giving sensitivities ranging from ~30 to 100 nm/RIU and a detection limit of 10^−4^ RIU. Capillaries with a high-index fluorescent silicon QD coating have also been developed for protein biosensing using biotin–neutravidin as a specific interaction model^106^.

Quantum dots are more photostable than their organic dye counterparts; they reach a high quantum yield of fluorescence and can emit light over a wide spectral range. However, they are not widely used for biosensing because of their weak solubility in water and toxic materials in their composition^80^. The latter problem can be solved by using a relatively new type of QD made of carbon, which opens the opportunity to explore so-called ‘green photonics’. Carbon QD WGM lasers have been recently demonstrated^107^.

### Inorganic perovskites

Semiconductor perovskites, or ABX3 materials, basically consist of a cubic unit cell with a large monovalent cation (A) in the centre, a divalent cation (B) on the corners, and smaller X- on the faces of the cube. The energy bandgap is directly related to the chemical structure of the perovskite, and its manipulation allows the full visible range to be covered in WGM microlasers. WGM lasing has been demonstrated in a number of perovskite structures with different shapes such as formamidinium lead bromide perovskite microdisks^108^, CsPbBr_3_ microrods^109^, and patterned lead halide perovskite microplatelets^110^. WGM lasers can also be fabricated using perovskites as quantum dots^34^. Similar to QDs, perovskites allow gradual tuning of the emission wavelength^111^.

Controllable fabrication of perovskite microlasers is challenging because it requires template-assisted growth or nanolithography. Zhizhchenko et al.^112^ implemented an approach for fabrication of microlasers by direct laser ablation of a thin film on glass with donut-shaped femtosecond laser beams. This method allows fabrication of single-mode perovskite microlasers operating at room temperature in a broad spectral range (550–800 nm) with Q-factors up to 5500.

Perovskite materials have a wide number of potential applications, including gas sensors^113^. Currently, the main problems with perovskites in WGM microlasers and sensors are their degradation in aqueous media and low photostability^108^. Some attempts to alleviate the water instability of perovskites, which mainly affects the structural and emission performance, include encapsulation in a SiO_2_ shell, with the resulting composite assembled into a tubular whispering-gallery microcavity^114^.

### Other prospective materials for WGM lasers

New materials for WGM microlasers and biosensors for which sensing has not yet been demonstrated have a bright future. Of special interest are 2D materials for WGM microlasers such as graphene, transition metal dichalcogenides (WS_2_, MoS_2_) and tungsten disulphide sandwiched between hexagonal boron nitride^115,116^. WGM single-mode lasing resonance was realised in submicron-sized ZnO rod-based WGM cavities with graphene^117^. Carbon-based materials are prospective materials for WGM lasers and biosensors due to their biocompatibility. In addition to WGM resonators doped with carbon quantum dots and graphene, a diamond WGM ‘cold’ resonator with a Q-factor of 2.4 × 10^7^ has been demonstrated^118^. Nanodiamonds including nitrogen vacancy centres coupled to disk resonators can be used for single-photon generation^119^, with prospects for quantum sensors.

The niche of new materials for biosensors is gradually being expanded. Another example is MXenes, which were recently found to have strong sensitivity enhancement for biosensing, gas sensing and humidity sensing due to their metallic conductivity, hydrophilic surface, large specific surface, and wide-band optical absorption. The experimental evidence supports the mechanism by which the characteristics of 2D MXene Ti_3_C_2_T_x_ can enhance the sensitivities of fibre optic biosensors and can be applied to the detection of most trace biochemical molecules^120–122^.

## Sensing with WGM microlasers in living cells and organisms

The application of WGM microlasers for in vivo sensing in cells and organisms is often limited to the use of biocompatible materials, geometries and dimensions that do not significantly affect the integrity of the target system. Ideally, the microsensor should not cause cellular stress. In this section, we review various micro- and nanoprobing approaches that are suitable for in vivo sensing. First, we discuss some of the most promising photonic techniques for biological micro- and nanoprobing, with a view to their use in WGM sensing. Then, we review the use of active WGM resonators for intracellular lasing and in vivo sensing applications.

### Single-cell micro- and nanoprobing

**Fig. 1:**
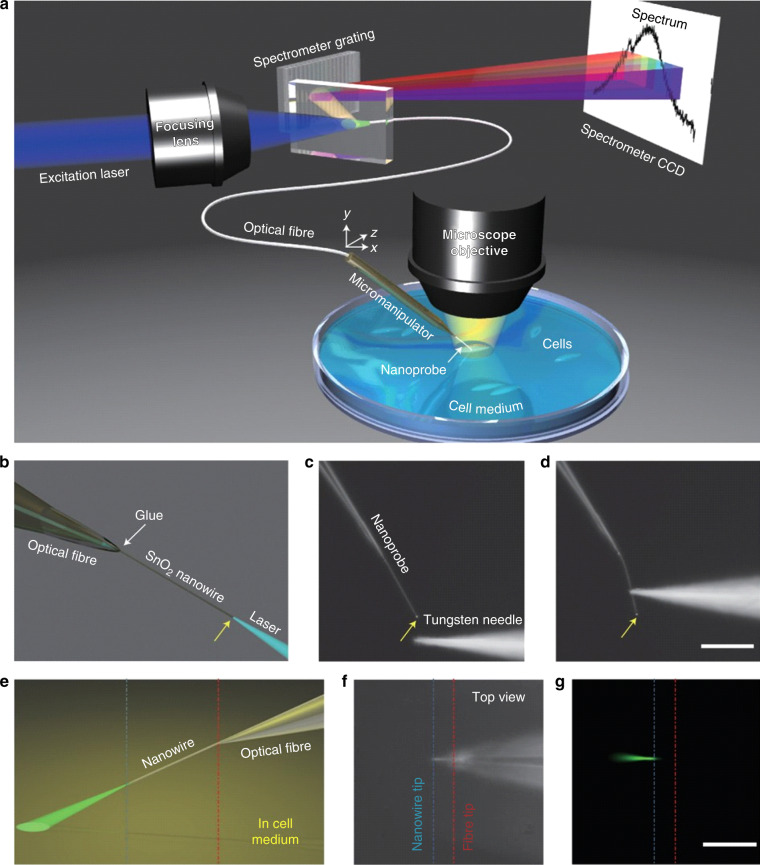
Example of nanoprobing using optical fibres combined with waveguiding materials tagged with fluorescent QDs. **a** Diagram of a nanowire-based cell endoscope; **b** diagram of a blue laser waveguided through a SnO_2_ nanowire attached to the tip of an optical fibre; **c**, **d** dark-field images of the nanowire-based cell endoscope before (**c**) and during (**d**) deformation by a tungsten needle to demonstrate its flexibility and robustness. The yellow arrows in **b**–**d** indicate the position of the nanowire tip where light emission into free space occurs. **e**–**g** Intensity emission profiles for an endoscope immersed in a cell culture medium, in which fluorescent proteins are illuminated with blue light from the nanowire tip; **e** diagram, **f** top-view dark-field image, and **g** top-view fluorescence image (a 442 nm longpass filter was applied). Scale bars 50 μm. Reproduced from ref. ^124^

Tagging and in vivo real-time sensing of physicochemical properties within single living cells is one of the main goals in biosensing. Despite the many challenges posed by the biocompatibility requirement on the microsensors used, several photonic micro- and nanoprobing techniques have already been successfully used for such applications^123^. An example of a successful approach relies on the modification of optical fibres with different sensing nanostructures, which ideally do not compromise cellular viability. Specifically, the insertion of a SnO_2_ nanowire waveguide tagged with fluorescent CdSe@ZnS streptavidin-tailored QDs (maximum emission at 655 nm) into the cell cytoplasm has been shown to enable in vivo endoscopy and controlled cargo delivery (Fig. 1)^124^. Optical pumping through a tapered fibre creates an evanescent field located in the region near the tip, where the nanowire is physically cleaved, which is thus suitable for local endoscopy and spectrometry.

**Fig. 2:**
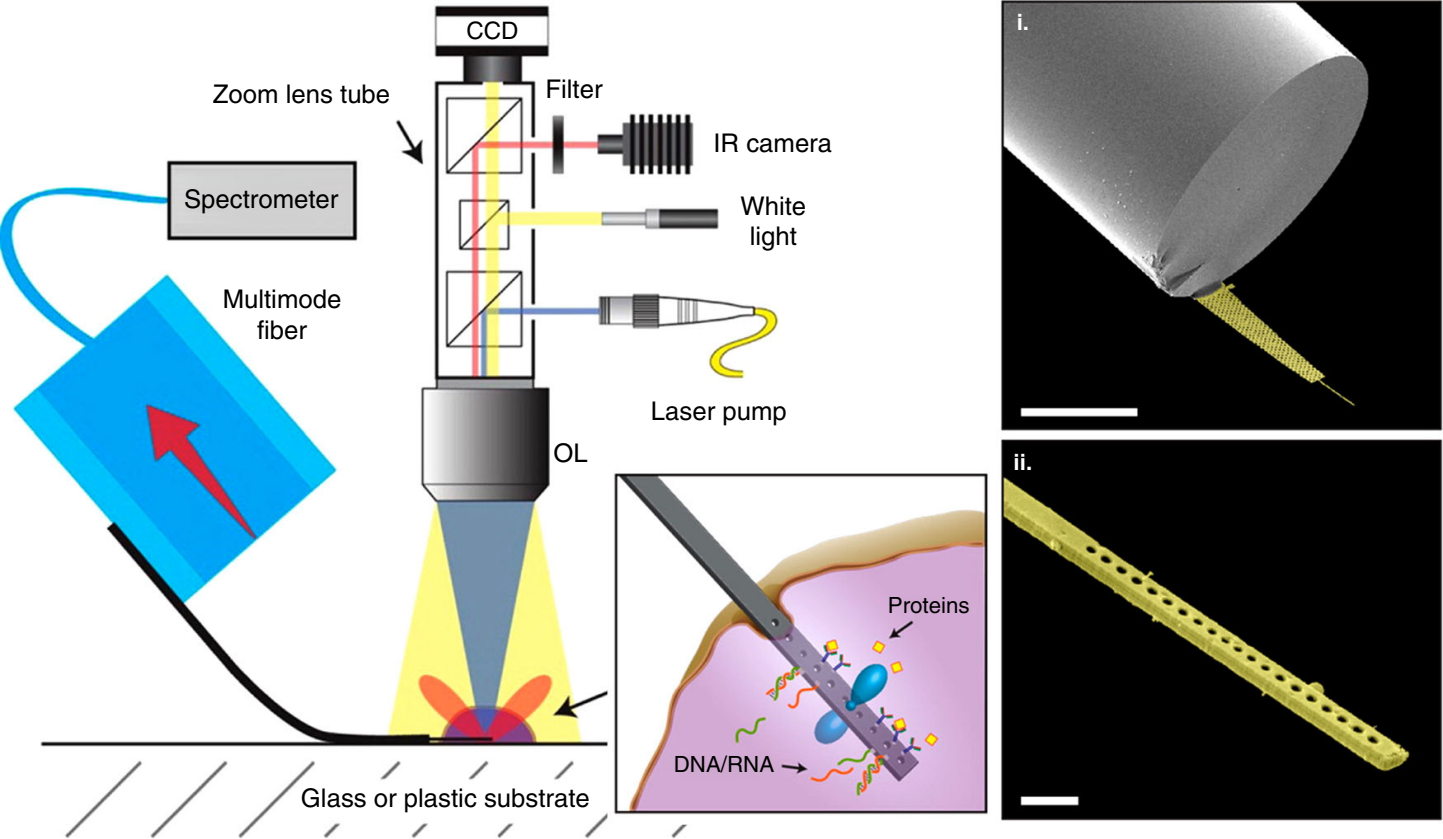
Schematic drawing of the optical setup used for remote readout with active GaAs semiconductor photonic crystal nanocavities doped with InAs QDs. The box depicts some of the potential sensing modes, e.g., label-free protein detection (left panels). False-colour SEM micrographs of an actual photonic crystal used for streptavidin (SA)-biotin-binding experiments (right panels i and ii)^125^

Another interesting example of a design that enables single-cell probing consists of the use of an active ‘nanobeam’ photonic crystal nanocavity constituted by a GaAs semiconductor doped with InAs QDs. These nanocavities have been shown to fulfil the biocompatibility requirement through experiments of internalisation using PC3 cancer cells in culture, in which normal cellular functions, such as migration and division, were maintained. Moreover, upon laser pumping, nanocavity spectra of the internalised probes were obtained, thus constituting the first reported example of active optical resonators in a biological environment, to the best of our knowledge. Shambat et al.^125^ showed the feasibility of remote optical readout sensing by performing in vitro protein sensing experiments for streptavidin (SA)-biotin binding, which opens the way for in vivo sensing using the described approach (Fig. 2)^125^. An appealing alternative would be the use of WGM ring resonators as active cavities instead of the crystal cavity geometry.

**Fig. 3:**
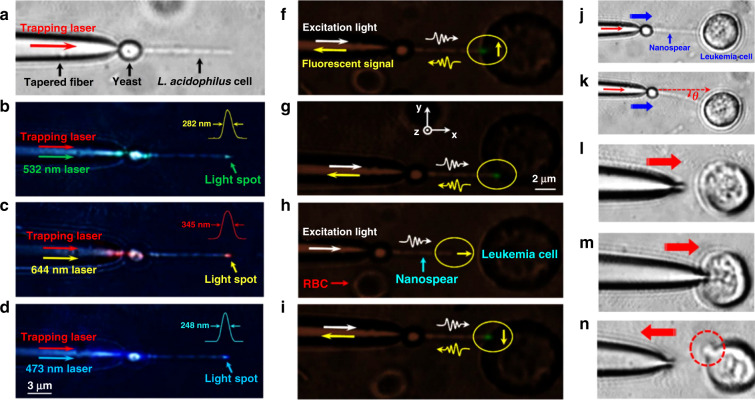
Microscopy images illustrating the construction of a living nanoprobe from yeast and bacteria single units, together with a sequence of actual use for probing a single leukaemia cell. **a** Bionanospear assembled on an optical fibre using single yeast and five *L. acidophilus* cells. **b**–**d** Dark-field images showing **b** 532 nm (green) light, **c** 644 nm (red) light and **d** 473 nm (blue) light propagating through the bionanospear and focused into subwavelength dimension spots (insets show line intensity profiles for the lateral direction in the spots). **f**–**i** Local fluorescence excitation/detection from a single leukaemia cell in human blood using the bionanospear. **f** Dark-field optical image of the bionanospear and leukaemia cell separated by a 3-μm gap. **j**–**n** Flexibility testing by pushing the bionanospear against the cell membrane of a leukaemia cell. Reproduced from ref. ^126^

On the same note, focusing on enhancing the biocompatibility, the use of so-called ‘living nanoprobes’ has recently been proposed^126^. This interesting example made use of in situ optical trapping at the tip of a tapered optical fibre, while the tapered fibre was inserted in a medium containing yeast, *L. acidophilus* and *leukaemia* cells (Fig. 3). Yeast cells were trapped on the tip upon external laser pumping, and self-assembly continued with the integration of *L. acidophilus* cells along the optical axis. Light was guided into the target (*leukaemia* cells), where localised fluorescence and optical signals were detected. These bionanospear probes demonstrate the value of biomimetic approaches towards single-cell sensing, with devices capable of concentrated illumination of subwavelength spatial regions. It is possible that the nanospear approach could be combined with WGM sensing by trapping a WGM microlaser at the tip of the fibre.

### WGM microlaser-based sensors in living systems: extra- and intracellular sensing

Photonic nanoprobes are limited to acting as waveguiding media, and the inclusion of active elements (preferably with high biocompatibility) is needed to meet the requirements of single-cell probing. The next logical and necessary step would be to generate stimulated emission in or by biological systems, rather than delivering laser radiation externally. In this regard, WGM microlasers can be used for tagging purposes and may provide valuable information on the functionality of a biological system by monitoring changes in resonator properties upon the application of a given stimulus.

Microlasers based on biomaterials can be further classified depending on whether the resonator configuration implies extra- or intracellular positioning. A remarkable example of an extracellular microlaser that uses Fabry–Pérot microcavities was presented by Gather and Yun in 2011^127^. In their work, the authors proposed a design based on living cells as a gain medium using *E. coli* cells that were previously modified so that they express green fluorescent protein (GFP)^128,129^. They used this device to demonstrate that the lasing properties from bacteria can be inherited by transmitting the capability to synthesise GFP upon cell division; this constitutes a crucial step towards large-scale self-sustained biological lasers. In another example, lasing amplification from live Venus protein-expressing *E. coli* bacterial cells was demonstrated to be feasible using WGM microdroplets^68^. Aside from using fluorescence proteins in extracellular microlasers, several approaches taking advantage of other biological structures were also recently reported, namely, assembly of feedback lasers using B2 vitamin-doped gelatine as a waveguide core^130^, use of nanostructured DNA films doped with fluorescent dyes^131^, fabrication of lasers based on chlorophyll-doped high Q-factor optofluidic ring resonators^89^, or even use of modified virus particles for lasing and biosensing^132^.

**Fig. 4:**
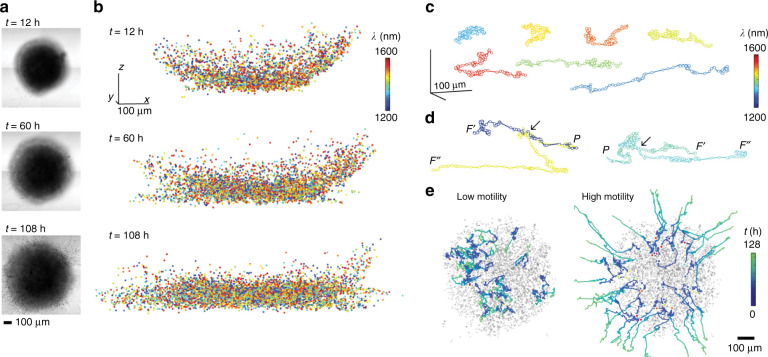
Illustration of the use of microdisk resonators (so-called laser particles or LPs) for cell tracking in a tumour spheroid upon internalisation. **a** Optical microscopy images of a tumour taken at different evolution times (12, 60 and 108 h). **b** Colour-coded tagged image (each colour corresponding to a given wavelength) of the spatial distribution within the tumour. **c** Mapped trajectories of selected microdisks, with a sampling rate of 1 h. **d** Trajectories corresponding to single parental cells (P) separating (marked by arrows) into two descendant cells (F′ and F′′). **e** Representative paths of cells classified according to measured average motility: high motility (top 25%) and low motility (bottom 25%). Initial positions are marked with a colour-coded circle depending on the wavelength; the path colour denotes the elapsed time over 128 h. Grey dots denote the positions of all microdisks at 12 h. Reproduced from ref. ^134^

Intracellular microlasers can open an entirely new avenue towards single-cell sensing, generating stimulated emission via biocompatible WGM cavities from within cells. One of the earliest examples in this regard was the use of polystyrene WGM microresonators (microspheres of 8–10 μm diameter) that allowed real-time sensing of biomechanical forces of endothelial living cells upon endocytosis^43^. Subsequently, using silica-coated microdisks as multiplexed microimaging probes, it was demonstrated that intracellular narrowband laser emission is feasible and enables tagging by spectral barcoding^133,134^. Furthermore, each studied cell type was able to internalise multiple microdisks, thus opening the possibility of multiplexed tagging of a large number of cells, allowing 3D tracking of individual cancer cells in a tumour spheroid and even motility measurements via long-term tracking over several days in mitotic 3T3 fibroblasts, as shown in Fig. 4.

An example of the internalisation of size-dispersed core-shell organic@silica microspheres, which can act as NIR WGM microresonators, was recently presented by Lv et al.^135^. The authors were able to distinguish and perform real-time tracking of 106 individual macrophage cells, even during the foaming process, which provided further insight into the dynamics of atherosclerosis, a major cause of cardiovascular diseases. NIR WGM microresonators are a promising example considering in vivo applications because they use nano/microjoule optical pumping and output light in the near-infrared wavelength range, thus considerably reducing the impact on cell physiology.

**Fig. 5:**
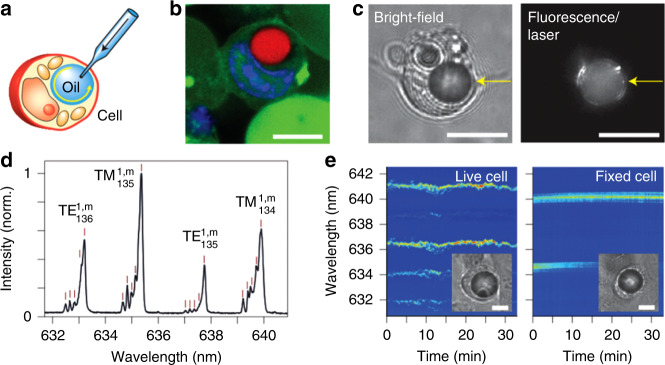
Illustration and microscopic images of the procedure followed to obtain oil microdroplets within single cells. **a** Schematic diagram of the injection of a PPE droplet into the cytoplasm of a cell. **b** Confocal fluorescence image of a cell containing a PPE droplet doped with Nile red dye (red). The nucleus of the cell can be seen in blue. **c** Bright-field (left) and laser output (right) images of a cell containing a PPE droplet. **d** Typical output spectrum of the lasing modes. **e** Time-lapse variation in the output spectrum for a live cell (left) and a dead cell fixed with formaldehyde (right). Adapted from ref. ^8^

Humar et al. have shown a simple and elegant way of generating nontoxic polyphenyl ether (PPE) oil droplets inside cells (Fig. 5)^63^. They used a microinjector connected to a glass micropipette with a 1 μm outer diameter and injected the oil into cells, which formed tiny droplets because of the immiscibility of the oil in the cell cytoplasm. The size of the injected droplets was controlled by the injection time, while the injection pressure was kept constant. Once the droplets were injected into the cells, a free-space coupling method, including an oil immersion objective, was employed for excitation of WGMs and collection of fluorescence^63^.

**Fig. 6:**
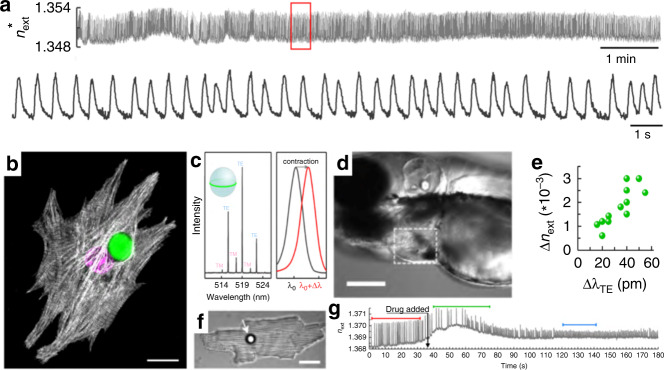
Real-time tracking of individual cell contractility through changes in the refractive index with both internalised WGM microbeads and extracellular microbeads in contact with the cell membrane for nonphagocytic adult cardiomyocytes. **a** Continuous single-cell monitoring with an intracellular microbead over 10 min (top) at 2 nJ/pulse, and magnification of the 20 s window indicated by the red rectangle (bottom). **b** 3D arrangement of myofibrils around microbeads in neonatal cardiomyocytes (CMs). Cell nucleus (magenta) and microlaser (green). **c** WGM spectrum of a microlaser showing multimode lasing (left). Illustration of the redshift in the lasing wavelength upon CM contraction (right). **d** Microlaser attached to the atrium of a zebrafish heart (3 days post-fertilisation); scale bar 200 µm. **e** Average refractive index change (Δη_ext_) between the resting phase (diastole) and peak contraction (systole) for 12 individual cells. **f** Extracellular microlaser (white arrow) on top of an adult CM. Scale bar 30 µm. **g** η_ext_ trace of a spontaneously beating neonatal CM during administration of 500 nM nifedipine (black arrow). Reproduced from ref. ^5^

In a very recent example dealing with intracellular WGM microlasers, it was demonstrated that spectral shifts of the WGMs caused by refractive index changes can be correlated with the contractility of an individual cardiac cell in living organisms^5^. Specifically, WGM microbeads were internalised and then acted as intracellular microlasers; their resonant emission wavelengths showed a redshift associated with cardiomyocyte contraction. By tracking the spectral position of the brightest lasing wavelength, a linearly approximated external refractive index (η_ext_) could be calculated, and the average η_ext_ changes showed a characteristic increase during cell contractions (Fig. 6a). Three-dimensional images of the studied cells demonstrate that microbeads are in direct contact with a dense network of myofibrils and thus so is the evanescent field of the laser mode (Fig. 6b). Since such proteins are involved in the contractile process, the origin of refractive index variations can be traced back to the fact that cell contractions significantly increase the protein density of the myofibrils. WGM microbead lasers can be readily internalised by different types of cardiac cells and even by zebrafish, for which cardiac contractility measurements were also performed. Moreover, these quantitative transient signals can be used to assess the effect of a calcium channel blocker drug (nifedipine), providing new insights into the mechanobiology of cardiac cells in general (Fig. 6g).

**Fig. 7:**
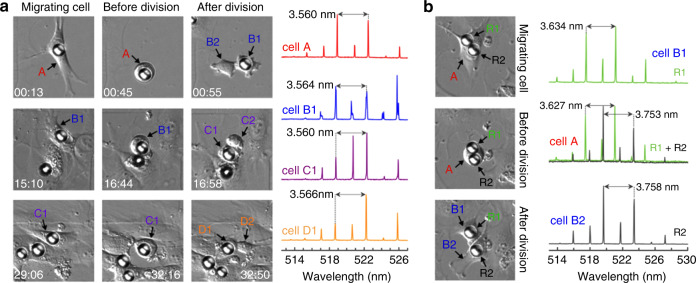
Long-term tracking of 3T3 fibroblast cells over several cell generations using internalised lipofectamine-treated polystyrene active microresonators. Mother cells are denoted A (red), and subsequent daughter generations are labelled B (blue), C (violet) and D (orange). **a** Left: differential interference contrast (DIC) images of a WGM laser within a migrating cell before, during, and after three cycles of cellular division. The time stamps indicated in the images are in hours:minutes and represent the period elapsed after the first lasing spectrum. Right: lasing spectra of the WGM resonator recorded during the migratory period, i.e., between cell division events. Arrows mark the free spectral range (FSR) between two neighbouring TE modes. **b** Left: tagging of both daughter cells (B1 and B2) from a mother cell carrying two intracellular lasers (R1 and R2). Right: lasing spectra of resonators inside the mother cell (centre, recorded separately for each resonator but plotted together) and after cell division (top/bottom). All DIC images show an area of 100 × 100 μm^2^. Reproduced from ref. ^136^

In general, approaches for conferring biocompatibility to microresonators make use of surface chemistry manipulation. For example, lipofectamine treatment applied to soft polystyrene active microresonators has been shown to facilitate endocytosis in four different types of cells. The feasibility of this approach has been demonstrated with the use of WGM microlaser-based cell tracking, which revealed broad compatibility with nervous system cells during division (N7 and SH-SY5Y cells), although such cells are generally believed to be nonphagocytic (Fig. 7)^136^.

**Fig. 8:**
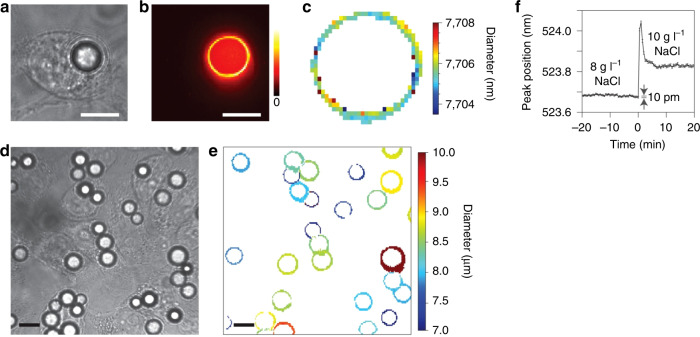
Intracellular microcavities used for tagging and intracellular sensing of relevant parameters in HeLa cells. **a** Bright-field image of a HeLa cell after internalisation of a polystyrene fluorescent bead. **b** Processed image of the cell in (**a**), where the false-colour intensity corresponds to the oscillating WGMs. **c** Calculated single-bead diameter map from confocal hyperspectral images corresponding to WGM output. **d**, **e** Images of bead-containing HeLa cells (**d**), and corresponding bead diameter map (**e**). **f** Time evolution of the resonant peak position for a bead inside a HeLa cell upon the addition of sodium chloride at *t* = 0; such exposure to a hypertonic solution produces cell volume shrinkage, which in turn causes the concentrations in the cytoplasm to vary, affecting the refractive index, which produces a shift in the peak wavelength. Scale bars 10 μm. Reproduced from ref. ^8^

Several biomaterials have been explored for fabrication of WGM intracellular lasers, such as dye-doped aptamer-modified silica microresonators^134^, microresonators doped with fluorescent dyes such as rhodamine B (RhB)^78^ or fluorescein^43^ fabricated from bovine serum albumin (BSA), and biopolysaccharides, among others. Highly biocompatible microcavities built from adipocytes from animal subcutaneous tissue have been demonstrated to enable laser emission under low-power pumping pulsed excitation. This is very suitable for the measurement of (in vivo) variations in the salt concentration in HeLa cells (Fig. 8)^8^. In addition, the conversion between B- and A-type starch structures has been monitored in soft starch granules doped with an organic dye for lasing emission^79^, which shows the potential of these devices for high-sensitivity sensing.

## Concluding remarks

The progress in biosensing with WGM microlasers is impressive. The many different ways that WGM microresonator materials and their multiple possible geometries can be combined and integrated with gain media result in a myriad of possible WGM microlaser devices that, as we have seen from this review, can have very exciting applications in biosensing. WGM microlaser sensors can be further optimised for the specific biosensing tasks at hand. For example, by functionalisation of the sensor with receptor molecules, one can achieve molecule-specific biodetection, and by integrating the sensors with microfluidics, one can achieve more controlled sample delivery and more reproducible data capture. The microgoblet WGM microlaser platform is an excellent example of this. It demonstrates reproducible and multiplexed detection of several different biomarkers via a single device integrated with microfluidics, where each WGM goblet sensor is functionalised with receptor-containing molecular inks. The WGM LCORR platform and WGM microdroplets and beads are other examples of versatile sensor platforms that can be tailored to meet a variety of different sensing needs, including detection of DNA, sensing of health-related protein markers and intracellular single-cell sensing of pH and forces.

This review shows that ongoing innovations in the fabrication and integration of microlasers with gain materials and lab-on-chip devices and the exploration of gain materials that provide more robust sensor operations or new functionalities (such as those based on polymers and MXenes, respectively) spur growing research activities on WGM microlaser sensors for real-world sensing applications. Developing these applications will require not only WGM devices with a robust and reproducible sensor response but also WGM sensors that operate in a highly multiplexed fashion, on chip or in solution, and that can be fabricated at low cost and for single use at the point of need.

There are a number of important challenges that need to be addressed before robust and clinically relevant in vitro and in vivo sensing applications of WGM microlaser sensors can become a reality. These challenges are mainly related to the lack of chemical stability of some of the cavity materials in water, the need for miniaturisation of the cavity so as not to perturb the biological cell or organism, the difficulty of biomolecular sensing in complex media where one encounters a host of unwanted background signals, and the difficulty of optical detection in highly scattering and absorbing biological media such as human tissue where WGM lasing at near-infrared wavelengths would be most desired. Methods are needed to discern the specific response of the WGM microlaser sensor to the binding of molecules from a background of resonance shifts due to temperature and bulk refractive index fluctuations. Referencing the measurements by comparing the frequency shifts of WGMs excited in the same microbead cavity may provide a way forward for achieving WGM microlaser sensing over prolonged time periods and under variable experimental conditions. For example, measuring relative frequency shifts in split-mode optoplasmonic WGM sensors has already been used as a sensing concept for highly sensitive detection of single molecules, and these measurements were mostly unaffected by changes in temperature and the host refractive index^137^.

Another of the outstanding challenges in WGM microlaser sensing is achieving high detection sensitivity at the level of single molecules. Passive WGM sensors have already demonstrated this ability; this has established them as an important platform to investigate the fundamentals of light-matter interactions, biomolecular structures and dynamics^138,139^. The WGM microlaser sensors can, in principle, become even more sensitive than their passive WGM counterparts. The difficulty lies in resolving the very small spectral shifts of the WGM laser lines on the order of ~10 MHz in single-molecule detection. A way forward may be the use of two laser lines for self-reference measurements within the same resonator and to reduce common mode noise, a concept that has, in part, been demonstrated for detection of very small ~15 nm nanoparticles^7^. Split-mode frequency shift detection with passive optoplasmonic WGM sensors that use plasmonic nanoparticles attached to WGM resonators has already enabled single-molecule detection. The passive ‘optoplasmonic’ WGM counterparts have demonstrated extremely high detection sensitivities^12^. The optoplasmonic WGM sensing concept has been used to detect even very small molecules, such as cysteamine (~77 Da), at attomolar concentrations, as well as single ions, such as single mercury and zinc ions, in aqueous solutions^140–142^. The application of the optoplasmonic split-mode single-molecule sensing concept^137^ to WGM microlasers should be explored to achieve single-molecule sensitivity. This approach may not only open up single-molecule sensing with WGM microlasers but also establish active WGM resonators as another important research platform to explore biomolecular interactions, their dynamics and the fundamentals of light-matter interactions in active optoplasmonic microcavities. The detection of single molecules inside a single cell using a WGM microlaser would be an exciting goal to pursue. Other important in vivo and in vitro diagnostic applications for ultrasensitive WGM microlasers to aim for include implantable sensors that detect health biomarkers and lab-on-chip devices that analyse biological samples, molecule-by-molecule, in ultrasmall (attolitre) detection volumes.

To conclude, WGM microlaser sensing is blossoming. This rapidly growing research area has the potential to address many of the most pressing biosensing challenges we are facing today. The coming decade will be the proving ground for biosensors such as WGM microlasers to deal with a myriad of global health and environmental concerns, including the emergence of new viruses and the detection of toxins in our water supplies. We need versatile sensors such as WGM microlasers to be best equipped to tackle these daunting challenges, i.e., by quickly and accurately detecting virus particles, health-related biomarkers and novel and harmful toxins in our drinking water.
